# First Reported Case of Donor Related Candida Endophthalmitis after Descemet Membrane Endothelial Keratoplasty

**DOI:** 10.2174/1874364101711010117

**Published:** 2017-06-19

**Authors:** Matthew Thompson, David Carli

**Affiliations:** 1 Tower Clock Eye Center 1087 W Mason St. Green Bay, WI, 54303, USA; 2 Medical Student Des Moines University, 3200 Grand Ave Des Moines, IA 50312, USA

**Keywords:** Candida, candida albicans, candida glabrata, fungal keratitis, descemet stripping endothelial keratoplasty, DMEK, corneal donor rim contamination

## Abstract

**Purpose:**

To report the first case of Candida donor to host transmission following descemet membrane endothelial keratoplasty (DMEK)

**Methods:**

A retrospective case report.

**Results:**

A patient underwent uneventful DMEK. Following surgery the donor rim was culture positive for Candida. The patient developed fungal endophthalmitis that was treated medically with multiple injections of voriconazole and amphotericin. Medical treatment was unable to clear the infection and removal of the donor material was required. Following removal the infection subsided.

**Conclusion:**

Candida interface keratitis and endophthalmitis can occur following DMEK and may be difficult to treat medically. Early removal of the donor material should be considered.

## INTRODUCTION

Endothelial keratoplasty (EK) has become the procedure of choice for corneal endothelial failure. Infections with fungal species are rare following corneal transplantation occurring at a rate of only 1.4 per 10,000 transplants performed [1]. The rate of fungal infections may be more common following EK than penetrating keratoplasty although the increase was not statistically significant [1].

Fungal infections primarily are from Candida species [2] and are difficult to eradicate, with 15 of 24 cases reported in the literature requiring surgical intervention, and 9 of 24 cases being treated medically [2-8].

At this time the literature contains reports of 24 cases of fungal endopthalmitis following descemet stripping endothelial keratoplasty (DSEK) [2]. Here we present the first published case of fungal endophthalmitis related to contaminated donor material following descemet membrane endothelial keratoplasty (DMEK) along with 22 months of follow up.

## MATERIALS AND METHODS

### Case Report:

A 75-year-old pseudophakic male with an acrylic intraocular lens in the bag presented to the clinic with a best-corrected visual acuity of 20/40 along with severe glare disability secondary to corneal guttata from Fuchs Endothelial Dystrophy. A DMEK procedure was performed on his right eye December 2, 2013 with pre-stripped tissue from the Cleveland eye bank stored in Optisol GS. The surgery was uncomplicated and included an intracameral injection of vancomycin at the conclusion of the case. The donor rim was sent for culture. The examination on postoperative day 1(POD#1) was unremarkable and the patient was started on moxifloxacin drops four times per day and prednisolone acetate 1% four times per day. On the evening after surgery the lab called to report that the donor rim was growing yeast. A discussion was held with the patient regarding the medical and surgical options and the patient was started on compounded Voriconazole 1% QID that he received on POD#2. On POD#4 the yeast was identified as Candida albicans, and the eye appeared quiet, the uncorrected acuity was 20/80. On POD #8 the eye became red and light sensitive. There was an increase in anterior chamber cells along with a cluster of five fluffy white keratic precipitates on the graft endothelium. The patient was referred to a retinal specialist and a tap and inject was performed with voriconazole. The patient was also started on oral voriconazole 200mg PO BID. A series of injections of intracameral voriconazole 100mcg were given on POD #10 and again on POD #12. The anterior segment became quiet and the uncorrected acuity improved to 20/40.

**Fig. (1):**
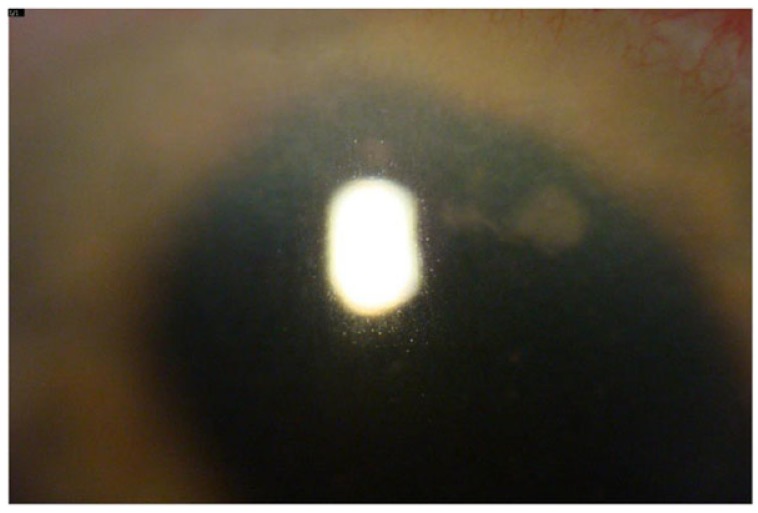
White fluffy keratic precipitates on the endothelial surface of the patients cornea 19 days after the initial DMEK surgery.

Nineteen days after surgery a cluster of five small white fluffy keratic precipitates again appeared on the DMEK graft endothelial surface (Fig. **1**). Visual acuity remained at 20/40 and the anterior chamber was not inflamed. The patient was taken to the OR and this area of the graft was removed and sent for culture, both Voriconazole 100mcg and Amphotericin 10mcg/0.1ml were injected into the anterior chamber. The prednisolone acetate drops were stopped and the patient was started on compounded cyclosporin 2%. Five days later the cultures from this procedure grew Candida glabrata, which was resistant to fluconazole.

34 days after surgery the eye again became light sensitive and red. The patient was seen by a retina specialist who performed an intravitreal injection of voriconazole along with a culture of the vitreous that yielded no growth. The eye became comfortable but multiple large white keratic precipitates reappeared on the DMEK graft endothelial surface and a decision was made to remove the DMEK graft in its entirety, which was done on POD#37. A series of injections of Voriconazole 100mcg and Amphotericin 10mcg/0.1ml were given at the time of removal and then every other day. After the 4^th^ injection, a culture was taken which again revealed Candida glabrata. A second series of 4 injections were then given after which aqueous was cultured twice and was negative for growth. Oral voriconazole was continued for 6 weeks after the first negative culture and topical voriconazole 6 times per day was continued for 3 months. During the series of injections, the patient did develop elevated intraocular pressure that was controlled medically. Additionally the cornea became very edematous and the eye was severely inflamed as there was no endothelium and no steroids were being used.

## RESULTS

4 months after removal of the DMEK donor the eye remained quiet and a DSAEK graft was placed. Recovery was uneventful but the patient did have interface haze that was obvious on slit lamp exam which obscured some details of the iris. The haze gradually has reduced over time. At 22 months post DSAEK the best spectacle corrected acuity was 20/60. He was stable on daily timolol and fluoromethalone and was not interested in pursuing penetrating keratoplasty to deal with the interface haze.

## DISCUSSION

A low percentage around 0.2% of DSEK grafts will have donor rims that are positive for fungus. [1,9] A significant percentage, perhaps as high as 67% of these cases, will go on to develop fungal endophthalmitis following EK surgery with fungal contaminated tissue [1-2]. EBAA data shows a trend towards increased rates of fungal infections following EK in comparison to PKP. [1] It is interesting to ask how the rate of infection will compare between DSEK and DMEK grafts. Although not statistically significant the EBAA has reported a trend towards an increased risk of fungal infections following EK in comparison to PKP. (Reference #1 Aldive). Theories as to the cause of this increased risk center around the presence of an interface in EK that is isolated from the anterior chamber. In comparison to a DSEK graft, a DMEK graft consists of a much smaller volume of transplanted tissue. In theory this may lead to a smaller inoculation of fungal units. However if the fungal contamination primarily rests on the surface of the donor tissue, then as the surface area of a DMEK and DSEK graft are nearly identical and both create an interface that is isolated from the anterior chamber, they should carry a similar rate of infectious risk.

In our case, the culture of the donor rim was identified as Candida Albicans. The subsequent cultures of the anterior chamber each revealed Candida Glabrata. It is reasonable to ask if the infection in this case is from the donor rim. Infections of the oral mucosa with Candida are better studied and it is common for Candida Albicans and Candida Glabrata to be present together. [10] It is much more likely that both organisms were present on the donor cornea but that only the Candida Albicans was identified by the microbiology lab than the alternative explanation: that the positive rim culture and subsequent endophthalmitis were two unrelated events.

Aggressive medical management with antifungal medications has been reported to allow the retention of some DSEK grafts. Another interesting question is whether medical treatment will be more successful in the case of DMEK grafts. Initially we believed that the smaller donor along with the decreased distance between the interface and the anterior chamber in a DMEK graft in comparison to a DSEK graft would increase the odds of successful medical treatment. 

In this first case however we were not successful in clearing the infection medically and surgery was required. Additional cases will need to be reported before any conclusions can be reached. Hence while treatment recommendations remain controversial, and more data is needed to reach firm conclusions, it is reasonable to consider early surgical intervention in the face of fungal endophthalmitis arising from a contaminated donor graft used for DMEK.

Supplementation of Optisol-GS storage medium with Amphotericin B or Voriconazole has been studied. [11-12] Both agents were effective in reducing the incidence of positive cultures. The EBAA has held the position that the low incidence of fungal infections does not warrant the routine use of antifungal medications in storage media.

 However case reports indicate a high rate of poor outcomes following fungal infections with the majority requiring further surgical intervention [2]. While it is outside of the scope of this report, a cost analysis would be a valuable study to determine the cost effectiveness of antifungal prophylaxis. Given the high morbidity of fungal infections we hope that the EBAA will continue to study the routine use of antifungal prophylaxis in the future.
